# Electrophysiological Substrate and Pulmonary Vein Reconnection Patterns in Recurrent Atrial Fibrillation: Comparing Thermal Strategies in Patients Undergoing Redo Ablation

**DOI:** 10.3390/jcdd12080298

**Published:** 2025-08-02

**Authors:** Krisztian Istvan Kassa, Adwity Shakya, Zoltan Som, Csaba Foldesi, Attila Kardos

**Affiliations:** 1 Gottsegen National Cardiovascular Center, 1096 Budapest, Hungary; zoltan.som@gokvi.hu (Z.S.); csaba.foldesi@gokvi.hu (C.F.); attila.kardos@gokvi.hu (A.K.); 2 Faculty of Medicine, Doctoral College of Semmelweis University, 1085 Budapest, Hungary; 3 Faculty of Medicine, Semmelweis University, 1085 Budapest, Hungary; adwityps@gmail.com

**Keywords:** atrial fibrillation, pulmonary vein isolation, mapping, pulmonary vein reconnection, radiofrequency, cryoballoon

## Abstract

Background: The influence of the initial ablation modality on pulmonary vein (PV) reconnection and substrate characteristics in redo procedures for recurrent atrial fibrillation (AF) remains unclear. We assessed how different thermal strategies—ablation index (AI)-guided radiofrequency (RF) versus cryoballoon (CB) ablation—affect remapping findings during redo pulmonary vein isolation (PVI). Methods: We included patients undergoing redo ablation between 2015 and 2024 with high-density electroanatomic mapping. Initial PVI modalities were retrospectively classified as low-power, long-duration (LPLD) RF; high-power, short-duration (HPSD) RF; or second-/third-generation CB. Reconnection sites were mapped using multielectrode catheters. Redo PVI was performed using AI-guided RF. Segments showing PV reconnection were reisolated; if all PVs remained isolated and AF persisted, posterior wall isolation was performed. Results: Among 195 patients (LPLD: 63; HPSD: 30; CB: 102), complete PVI at redo was observed in 0% (LPLD), 23.3% (HPSD), and 10.1% (CB) (*p* < 0.01 for LPLD vs. HPSD). Reconnection patterns varied by technique; LPLD primarily affected the right carina, while HPSD and CB showed reconnections at the LSPV ridge. Organized atrial tachycardia was least frequent after CB (12.7%, *p* < 0.002). Conclusion: Initial ablation strategy significantly influences PV reconnection and post-PVI arrhythmia patterns, with implications for redo procedure planning.

## 1. Introduction

Pulmonary vein isolation (PVI) is a well-established therapeutic approach for atrial fibrillation (AF), aiming to eliminate arrhythmogenic triggers originating from the pulmonary veins (PVs) [1,2]. While PVI effectively reduces AF burden, recurrence remains common, often due to pulmonary vein reconnection (PVR) [3]. The durability of PV isolation and the pattern of residual conduction can vary based on the initial ablation strategy, lesion formation characteristics, and tissue response. These factors suggest that the choice of ablation modality not only impacts long-term outcomes but may also influence the mechanisms of arrhythmia recurrence and the optimal strategy for redo ablation.

Several energy sources and technologies are available for PVI, with radiofrequency (RF), cryoballoon (CB), and pulsed-field ablation (PFA) being the most widely used [4]. RF ablation can be delivered using different protocols, such as low-power, long-duration (LPLD) and high-power, short-duration (HPSD) approaches [5]. RF energy is applied point-by-point, producing thermal lesions via resistive heating and conductive tissue damage [6]. In contrast, CB ablation employs a single-shot cryogenic application, inducing circumferential necrosis around the PV ostia through tissue freezing [7].

Although individual ablation strategies have been studied extensively, comparative data on their long-term effects—particularly regarding PV reconnection patterns and redo PVI outcomes—remain limited. A better understanding of how initial ablation modalities affect lesion durability and atrial substrate remodeling is essential for refining ablation techniques and improving procedural success.

This study aimed to investigate the influence of initial PVI modality—AI-guided LPLD RF, HPSD RF, or second-/third-generation CB—on remapping findings during redo ablation for recurrent atrial arrhythmias. Specifically, we analyzed PV reconnection patterns and left atrial substrate characteristics using high-density electroanatomic mapping, to provide insights that may guide the optimization of initial ablation strategies and improve outcomes for patients requiring repeat procedures.

## 2. Materials and Methods

### 2.1. Study Population

We retrospectively screened all patients referred for redo ablation due to recurrent atrial arrhythmia following an initial PVI between January 2015 and June 2024, using our institutional ablation registry. Patients were eligible if high-density electroanatomic mapping was performed during the redo procedure using multielectrode mapping catheters. Patients with atypical atrial flutter as the index arrhythmia or incomplete procedural data were excluded. All procedures were conducted in accordance with the Declaration of Helsinki. Informed consent was obtained from all patients prior to participation.

### 2.2. Mapping and Assessment of Pulmonary Vein Reconnection

**Figure 1:**
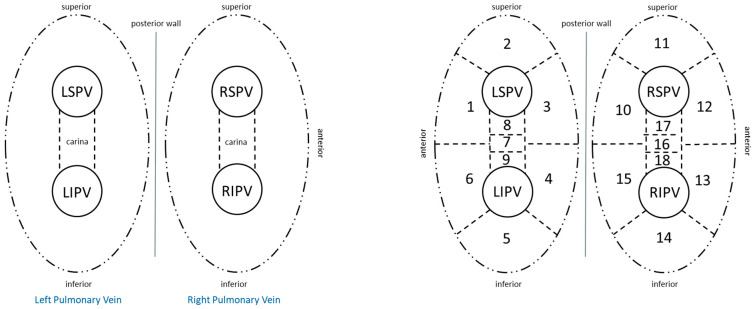
Eighteen-segment model of the left atrium and pulmonary veins used for mapping and registration of conduction gaps during redo ablation. Abbreviations: LSPV, left superior pulmonary vein; LIPV, left inferior pulmonary vein; RSPV, right superior pulmonary vein; RIPV, right inferior pulmonary vein.

Following femoral venous access, transseptal puncture was performed under fluoroscopic and/or intracardiac echocardiography guidance. After transseptal access, a multipolar mapping catheter (Pentaray or Octaray, Biosense Webster, Diamond Bar, CA, USA) was advanced into the LA, and high-density electroanatomic mapping was performed using a 3D mapping system (CARTO, Biosense Webster, Diamond Bar, CA, USA). Mapping was conducted in sinus rhythm or during coronary sinus pacing in cases of persistent AF to ensure accurate assessment of conduction gaps. PVR was assessed by systematically interrogating each PV antrum for the presence of local electrograms. Reconnection was confirmed by the presence of spontaneous or pacing-induced PV potentials, along with differential pacing maneuvers (especially from the left atrium appendage, LAA) to distinguish far-field signals from true PV potentials. Each PV was anatomically segmented using a standardized 18-segment model comprising the antral and ostial regions of all four PVs, including anterior, posterior, superior, inferior, and carinal zones (see schematic drawing for segmental registration in Figure 1). Reconnection sites were annotated in the electroanatomic map to allow for quantitative and spatial comparison between the different index ablation modalities. Only unequivocal reconnection sites with stable electrogram capture and consistent pacing response were included in the final segment-based reconnection analysis. Importantly, the investigators responsible for reconnection pattern registration were blinded to the index ablation technique to minimize bias.

### 2.3. Redo Ablation Procedure

**Figure 2:**
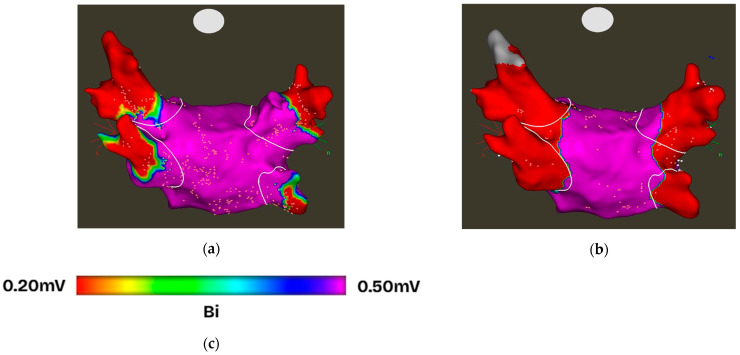
Bipolar voltage map of a patient with recurrent atrial fibrillation following index low-power long-duration (LPLD) radiofrequency (RF) pulmonary vein isolation (PVI). (**a**) Pulmonary vein reconnection (PVR) is evident in all PVs, with high-voltage signals (>0.5 mV) over the posterolateral (PL) ostia. (**b**) Bipolar voltage map after segmental redo RF ablation demonstrates successful reisolation of all PVs. (**c**) Color scale representing bipolar voltage amplitude from 0.20 mV (red) to 0.50 mV (purple). Abbreviations: LPLD, low-power, long-duration; PV, pulmonary vein; PVR, pulmonary vein reconnection.

All redo ablation procedures were performed using radiofrequency (RF) ablation. Segments exhibiting PV reconnection were targeted for reisolation (Figure 2). In patients with all PVs remaining durably isolated, but with persistent AF, additional substrate modification was performed via posterior wall isolation, utilizing either a posterior box or posterior wall debulking strategy based on operator discretion (see an example in Supplementary Figure S1).

### 2.4. Index Ablation

The initial ablation modality was retrospectively identified based on detailed procedural reports and electroanatomic data, including Visitag™ module parameters and ablation log data recorded by the electroanatomic mapping system (CARTO, Biosense Webster, Diamond Bar, CA, USA). Patients were categorized into three groups according to the energy source and lesion formation strategy used during their index PVI procedure:

1. AI-guided low-power, long-duration (LPLD) RF ablation;
2. AI-guided high-power, short-duration (HPSD) RF ablation;
3. Second- or third-generation cryoballoon (CB) ablation.

**Figure 3:**
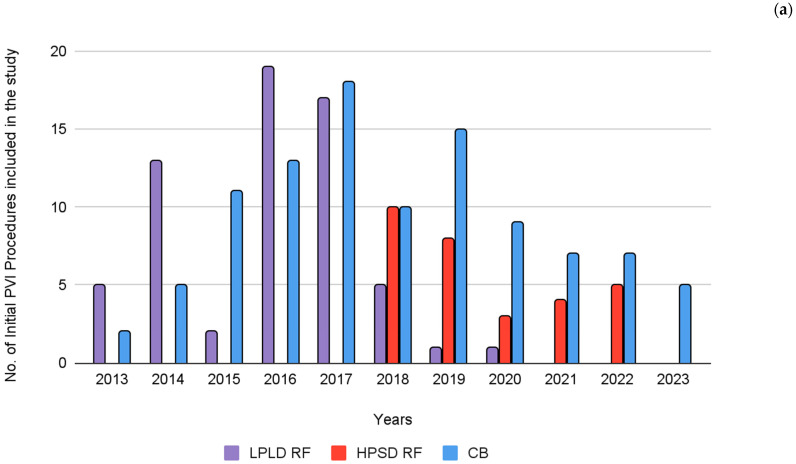

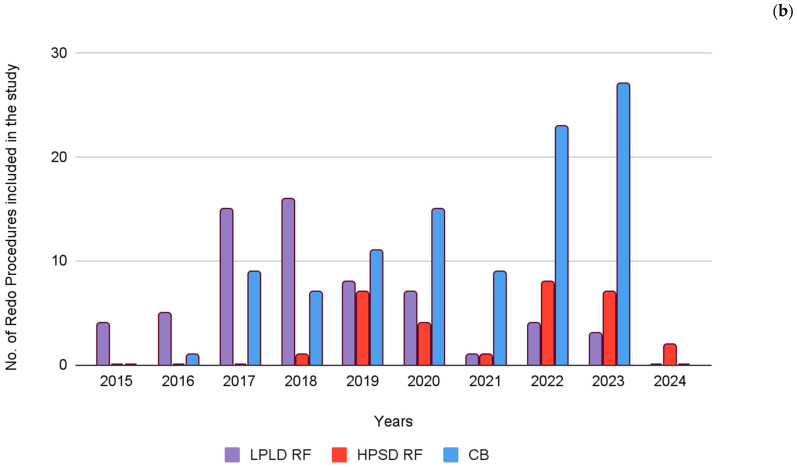
Timeline of procedures performed over the years. Panel (**a**) Index PVI procedures using low-power, long-duration radiofrequency (LPLD RF), high-power, short-duration radiofrequency (HPSD RF), and cryoballoon (CB). Panel (**b**) Redo PVI procedures following initial ablation with LPLD RF, HPSD RF, or CB.

Figure 3 illustrates the distribution of cases across the three ablation strategies—LPLD RF, HPSD RF, and CB used throughout the study period.

### 2.5. LPLD Radiofrequency Ablation

In the LPLD group, ablation was performed using a Thermocool SmartTouch catheter (Biosense Webster Diamond Bar, CA, USA) in power-controlled mode, delivering 30–35 W of energy. Lesion formation was guided by the AI, an integrated metric incorporating contact force, power, and time [8]. Target AI values were set at 500–550 for anterior wall segments and 400–450 for posterior wall sites. A point-by-point approach was employed to achieve continuous circumferential ablation around each PV, aiming for complete isolation.

### 2.6. HPSD Radiofrequency Ablation

HPSD ablation was performed using either the Thermocool SmartTouch or QDot Micro catheter (Biosense Webster Diamond Bar, CA, USA). As in the LPLD group, lesion formation was guided by AI, with target values of 500 for posterior wall segments and 550 for anterior regions [9]. The QDot Micro catheter operated in Q-mode, delivering 50 W of power with real-time temperature monitoring and optimized energy delivery, enhancing both safety and lesion consistency. When using the Thermocool SmartTouch catheter, ablation on the posterior wall was approached with particular caution due to esophageal proximity. In these areas, lesion duration and catheter position were carefully adjusted based on impedance drop, temperature feedback, and local electrogram attenuation to reduce the risk of collateral injury.

### 2.7. Cryoballoon Ablation

Cryoballoon ablation was performed using second- or third-generation Arctic Front Advance or Arctic Front Advance Pro balloon catheters (Medtronic, Inc., Minneapolis, MN, USA). After single transseptal puncture and advancement of a steerable sheath into the left atrium, a 28 mm cryoballoon was advanced and positioned at each PV ostium using the Achieve mapping catheter for both guidance and real-time PV electrogram monitoring. Adequate vein occlusion was confirmed by contrast injection. Cryothermal energy was delivered for 180–240 s per application. Two applications per vein were applied on the left PVs, and one application per vein on the right PVs, with continuous phrenic nerve pacing during right-sided ablation to monitor for phrenic nerve injury.

### 2.8. Procedural Endpoints

In all index procedures, the procedural endpoint was complete electrical isolation of all pulmonary veins, confirmed by the demonstration of both entrance and exit block. Adenosine testing was not performed. No posterior wall isolation or additional substrate modification was performed during the index ablation.

### 2.9. Data Collection and Outcomes

Demographic, procedural, and clinical data were collected from the institutional database. Primary outcomes included the prevalence of pulmonary vein (PV) reconnections at redo ablation, the number of reconnected segments per patient, and the segmental distribution of PV reconnection based on an 18-segment left atrial model. Secondary outcomes included the presence of organized atrial tachycardia (AT) during redo mapping, 12-month arrhythmia-free survival following redo ablation, and a comparison of procedural success between patients with recurrent atrial fibrillation (AF) versus AT.

### 2.10. Statistical Analysis

Continuous variables were expressed as mean ± standard deviation or median (interquartile range), depending on data distribution. Categorical variables were reported as frequencies and percentages. Comparisons between ablation modality groups were made using ANOVA or Kruskal–Wallis tests for continuous variables and chi-square or Fisher’s exact tests for categorical variables, as appropriate. A multivariate logistic regression analysis was performed to identify independent predictors of pulmonary vein reconnection, the occurrence of organized atrial tachycardia at redo, and the acute success rate of the redo ablation procedure. A two-tailed *p*-value < 0.05 was considered statistically significant. Statistical analysis was performed using SPSS (version 30, IBM Corp, Armonk, NY, USA) and R (version 4.3.1. R Foundation for Statistical Computing, Vienna, Austria).

## 3. Results

### 3.1. Baseline Demographics

A total of 195 patients undergoing redo ablation with high-density mapping were included: 63 had prior LPLD RF ablation; 30 had HPSD RF; and 102 had undergone CB PVI.

The mean age was significantly lower in the CB group (61.88 ± 2.14 years) compared to LPLD RF (65.6 ± 2.56 years, *p* < 0.05), with no significant age difference between the RF groups. The male proportion did not differ significantly across groups (LPLD RF: 49.2%, HPSD RF: 43.3%, CB: 38.3%). Paroxysmal AF was present in 55.6%, 53.3%, and 48.0% of patients in the LPLD RF, HPSD RF, and CB groups, respectively (*p* > 0.05). No significant intergroup differences were observed regarding AF duration, body mass index (BMI), left ventricular ejection fraction (LVEF), LA diameter, or comorbidities. Detailed baseline characteristics are presented in Table 1.

### 3.2. Remapping Outcomes

**Figure 4:**
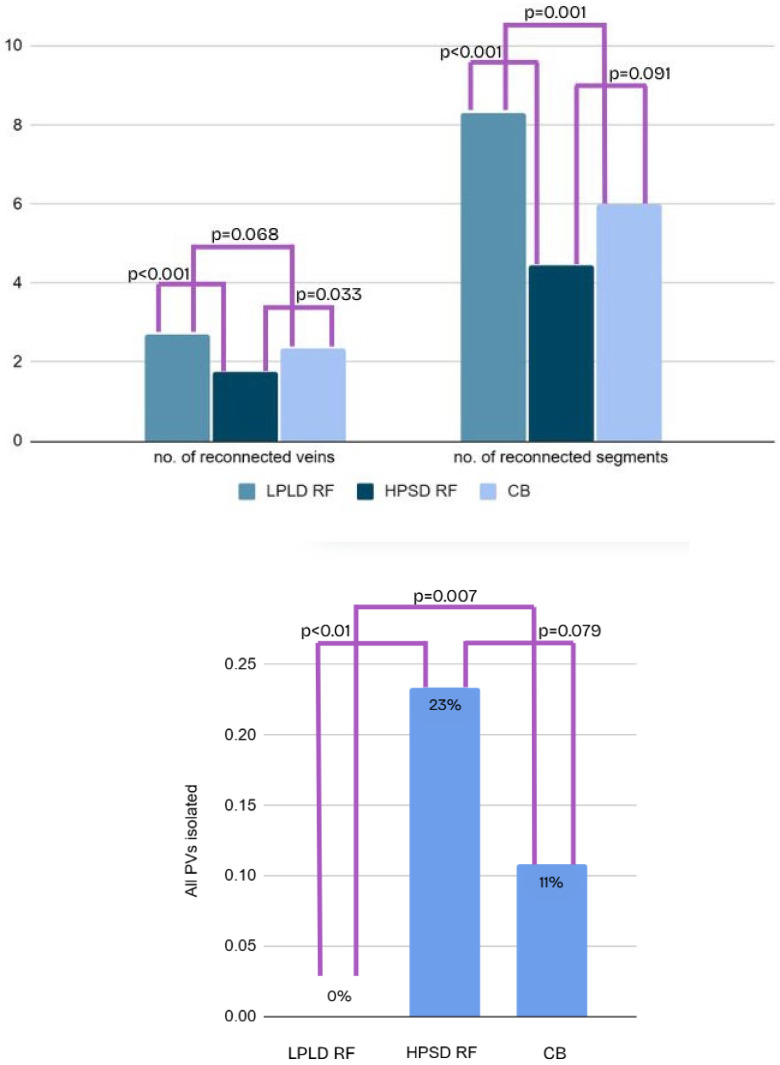
Remapping findings based on the initial pulmonary vein isolation (PVI) technique. The figure summarizes pulmonary vein reconnection (PVR) rates, the average number of reconnected segments per patient, and the number of patients with recurrent atrial fibrillation (AF) in whom all pulmonary veins remained isolated at redo. Data are grouped by initial ablation modality: low-power, long-duration radiofrequency (LPLD RF); high-power, short-duration radiofrequency (HPSD RF), and cryoballoon ablation (CB). Segmental reconnection was most extensive following LPLD RF, whereas durable isolation of all PVs was most frequently observed after HPSD RF.

Redo procedural characteristics are shown in Table 2. Complete PVI at the time of redo ablation was confirmed in none of the patients who had initially undergone LPLD RF ablation, in 23.3% of those treated with HPSD RF (*p* < 0.01 vs. LPLD RF), and in 10.1% of those who had received CB ablation (*p* = 0.079 vs. HPSD RF). The number of reconnected segments was highest in the LPLD RF group, with an average of 8.3 ± 1.1 segments, followed by the CB group with 6.0 ± 0.9, and the lowest number was observed in the HPSD RF group with 4.5 ± 1.5 segments. These differences were statistically significant across all group comparisons (*p* < 0.05, Figure 4).

Distinct patterns of segmental reconnection were identified based on the initial ablation modality. In the LPLD RF group, the right carina was most frequently reconnected, and the most commonly affected vein was the right inferior pulmonary vein (RIPV). In contrast, patients with prior HPSD RF or CB ablation predominantly showed reconnection at the ridge and anterior wall of the left superior pulmonary vein (LSPV), which was also the most frequently reconnected vein in these groups. Multivariate logistic regression analysis demonstrated that only the index ablation strategy remained a significant independent predictor of pulmonary vein reconnection at remapping (*p* = 0.0069).

**Figure 5:**
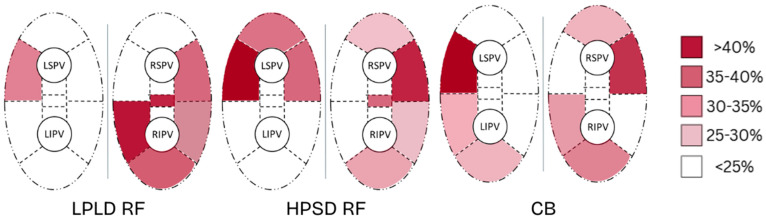
Segmental distribution of pulmonary vein conduction gaps according to the initial ablation modality: low-power, long-duration radiofrequency (LPLD RF); high-power, short-duration radiofrequency (HPSD RF); and cryoballoon (CB). The figure illustrates reconnection frequency across the 18-segment left atrial model. LPLD RF was associated with widespread segmental reconnections, particularly involving the right carina. HPSD RF showed fewer gaps overall, with a tendency toward localized reconnections. CB demonstrated a distinct reconnection pattern with predominant gaps in the anterior and ridge segments.

**Figure 6:**
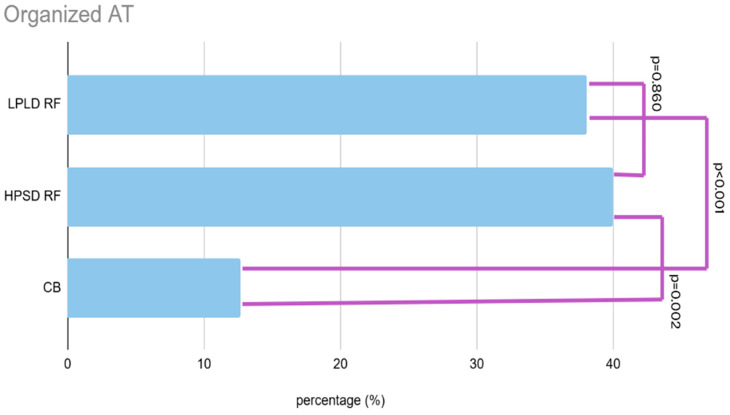
Incidence of organized atrial tachycardia according to the initial PVI modality: LPLD RF, HPSD RF, and CB. Organized atrial tachycardia was significantly less frequent following CB-based PVI compared to RF groups, suggesting a potential link between ablation technique and arrhythmia phenotype at recurrence.

The occurrence of non-AF, organized atrial tachycardia was significantly less frequent in the CB group, affecting 12.7% of patients, compared to 38.1% in the LPLD RF group and 40.0% in the HPSD RF group (*p* < 0.002). A detailed comparison of reconnection patterns is presented in Figure 5 and Table 3, and the graphical comparison of non-AF post PVI atrial tachycardia is shown in Figure 6.

### 3.3. Clinical Outcomes: Complications and Success Rates

The overall complication rate was comparable across all groups: 1.6% in the LPLD RF group, 3.3% in the HPSD RF group, and 1.9% in the CB group (*p* > 0.05). Reported complications included inguinal hematoma, access-site bleeding, and one instance of cardiac tamponade.

The success rate of redo ablation in patients with organized atrial tachycardia (non-AF AT) as the recurrent arrhythmia was 73.5%, compared to 82.2% in patients with recurrent atrial fibrillation (*p* = 0.22). Redo procedures tended to be less successful in patients with persistent atrial fibrillation (74.7%) compared to those with paroxysmal AF (83%), although this difference was also not statistically significant (*p* = 0.215).

**Figure 7:**
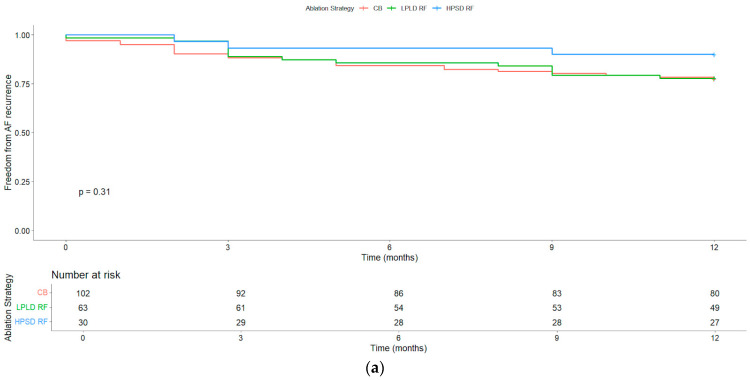

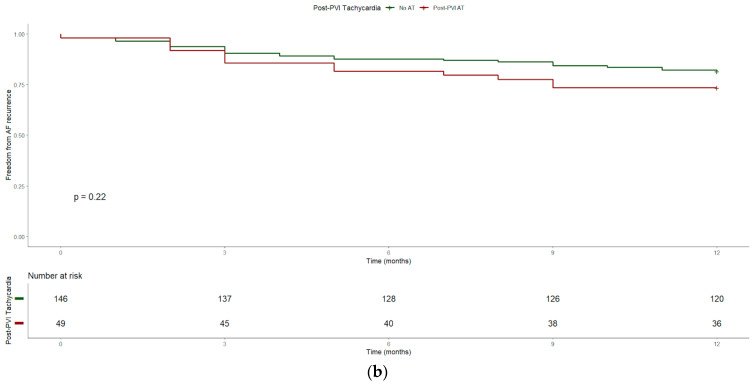
Kaplan–Meier arrhythmia-free survival at 12 months following redo ablation: (**a**) stratified by initial pulmonary vein isolation (PVI) strategy: low-power, long-duration radiofrequency (LPLD RF); high-power, short-duration radiofrequency (HPSD RF); and cryoballoon (CB); (**b**) recurrent atrial fibrillation (AF) versus organized atrial tachycardia (AT). Survival curves demonstrate comparable outcomes across groups, with no statistically significant differences observed.

At 12-month follow-up, the highest arrhythmia-free survival rate was observed in the HPSD RF group (90%), followed by the LPLD RF group (77.8%) and the CB group (76.5%). However, these differences did not reach statistical significance (*p* > 0.05). See Figure 7 for arrhythmia-free survival curves.

## 4. Discussion

### 4.1. Key Findings

This study provides novel comparative insights into the long-term electrophysiological outcomes of different first-line ablation strategies in patients undergoing redo procedures for recurrent atrial arrhythmias. To our knowledge, this is among the first remapping studies comparing three distinct first-line PVI modalities using a standardized high-resolution segmental mapping approach.

A key finding is that complete PVI at the time of redo was most commonly observed in patients who had initially undergone HPSD RF ablation, suggesting this approach yields a more durable lesion set over the long term. In contrast, none of the patients with prior LPLD RF ablation exhibited durable isolation, underscoring the limitations of this technique in achieving sustained conduction block. The CB group showed intermediate durability.

Another notable observation was the significantly lower incidence of organized atrial tachycardias (non-atrial fibrillation, non-AF AT) in the CB group compared to both RF cohorts. This may reflect more homogeneous and contiguous lesion formation with CB, potentially minimizing pro-arrhythmic conduction gaps and reducing the development of macro-reentrant or focal atrial tachycardias.

Distinct reconnection patterns emerged across ablation modalities—particularly, reconnections at the RIPV/right carina in the LPLD RF group and at the left superior PV ridge and anterior wall in both CB and HPSD RF groups. These site-specific vulnerabilities likely reflect regions of thicker atrial tissue or catheter instability and emphasize a critical lesson in thermal ablation: certain anatomical areas consistently require more deliberate energy delivery or adjunctive strategies to achieve durable isolation, regardless of the ablation technology used.

### 4.2. Impact of Initial Ablation Modality on PVR

Prior studies have sought to characterize PVR patterns using detailed electroanatomic mapping, with the aim of optimizing lesion formation strategies following RF or CB ablation. In the ICE Re-Map study, Chen et al. demonstrated that extending the CB freeze duration from 180 to 240 s significantly improved lesion durability at the left-sided pulmonary veins [10]. Similarly, in a randomized trial incorporating a protocol-mandated repeat procedure, Sørensen et al. reported high overall isolation rates; however, the distribution of reconnection sites differed markedly between energy sources. Reconnection following conventional RF ablation was predominantly localized to the carina and adjacent segments, whereas CB-related gaps were more heterogeneously distributed across all four pulmonary veins [11].

During the inclusion period, there was a gradual shift in the use of mapping catheters, with increasing adoption of the OctaRay catheter alongside continued use of PentaRay. In parallel, periodic upgrades to the electroanatomic mapping system were implemented. Advances in ablation catheter technology—such as the introduction of ThermoCool SmartTouch™, QDot Micro™, and successive cryoballoon generations (2nd and 3rd) —were also progressively incorporated into clinical practice. Importantly, all PVI procedures were performed following a consistent and standardized protocol, which remained unchanged throughout the study period, including the application of rigorous lesion index criteria. Multivariate logistic regression analysis revealed that the index ablation modality was the only significant independent predictor of pulmonary vein reconnection at redo.

LPLD RF ablation may suffer from suboptimal lesion contiguity or transmurality, especially at anatomically complex sites such as the carina or thicker posterior segments [12]. However, because mapping in this study was performed only in patients with recurrence, it is possible that other patients had durable PVI—unlike studies with mandatory remapping at 3 months [13,14]. HPSD RF ablation shows superior lesion durability, likely due to deeper and more homogeneous lesion formation [15]. CB ablation reconnections, especially at the LSPV ridge, may reflect anatomical challenges in achieving consistent balloon-to-tissue contact [16]. Anatomical considerations such as thick tissue and potential epicardial connections at the carina, along with concerns related to the proximity of the posterior wall to the esophagus, may contribute to reconnections [17]. Ridge and anterior wall segments are often thicker, and catheter instability may be under-addressed due to balloon fit limitations [18].

### 4.3. Post-PVI Organized Atrial Tachycardia

The lower rate of non-AF organized atrial tachycardias in CB patients may reflect a more uniform lesion set with less collateral atrial damage. In contrast, RF ablation, particularly if fragmented or incomplete, can create pro-arrhythmic conduction channels leading to organized atrial tachycardias. CB ablation, with its larger lesion size and less need for substrate modification, may help mitigate this risk [19]. It is also noteworthy, that our study protocol included remapping following the redo PVI procedure that may further improve outcomes [20].

### 4.4. Redo Procedure Success Rates

Redo success rates did not differ significantly across initial ablation strategies, with relatively higher success observed following HPSD RF and CB ablation compared to LPLD RF. Challenging anatomical sites such as the carina and ridge consistently showed higher reconnection rates. Moreover, redo ablation for organized atrial tachycardia is often more technically demanding than for recurrent AF due to the complexity of identifying the arrhythmia circuit, but success rates appear comparable [21].

### 4.5. Clinical Implications for Patient Selection for Redo

These findings underscore the value of a personalized approach to redo ablation, whereby the choice of mapping and treatment strategy is informed by the characteristics of the initial ablation modality. This study advances the field in several important ways. First, by including patients who underwent three distinct first-line ablation strategies—LPLD RF, HPSD RF, and cryoballoon ablation—it provides a unique comparative perspective on long-term lesion durability. Second, the use of a standardized segmental reconnection map facilitates consistent and reproducible analysis of reconnection patterns across techniques. Third, the identification of modality-specific durability and reconnection profiles allows for more strategic procedural planning, including tailored mapping strategies, improved time efficiency, and optimized use of resources in the electrophysiology lab. In addition, the consideration of silent pulmonary veins—those not showing reconnection despite arrhythmia recurrence—remains critical during redo procedures, particularly when symptom recurrence is due to non-PV triggers or macroreentrant atrial tachycardias [22].

### 4.6. Limitations

Several limitations should be acknowledged. First, this is a single-center study, which may limit the generalizability of the findings to other institutions with different ablation protocols or operator experience. Second, although ablation modality groups were well-defined, the non-randomized, observational design may introduce selection bias, particularly regarding operator choice of energy source and technique. Third, technological advancements over time (e.g., evolving RF catheters, mapping systems, and lesion index targets) may have influenced outcomes, especially in the LPLD RF group, which included procedures performed earlier in the study period. Fourth, reconnection patterns were assessed only at the time of redo, and not by standardized remapping in all patients’ post-index ablation, potentially underestimating transient or recovering conduction. Finally, although the reconnection map was standardized, its interpretation may still be subject to inter-operator variability in electroanatomic annotation and signal quality.

## 5. Conclusions

This study provides novel comparative insights into long-term electrophysiological outcomes following three distinct initial ablation modalities in patients undergoing redo procedures for recurrent atrial arrhythmias. HPSD radiofrequency ablation demonstrated the highest rate of durable PVI at redo, whereas LPLD RF showed the least durability. Cryoballoon ablation was associated with a lower incidence of organized atrial tachycardias, likely due to more uniform lesion formation.

By applying a standardized segmental reconnection map and accounting for the initial ablation modality, this study highlights the value of a personalized redo strategy. Tailoring energy delivery and lesion creation based on known reconnection patterns in specific segments may enhance procedural efficiency and, importantly, improve long-term rhythm outcomes in this complex patient population.

## Figures and Tables

**Table 1:** Patient Demographics.

Parameter	LPLD RF (63)	HPSD RF (30)	CB (102)	*p*-Value (LPLD vs. HPSD)	*p*-Value (HPSD vs. CB)	*p*-Value (LPLD vs. CB)
Age (years)	65.6 ± 2.56	64.43 ± 4.37	61.88 ± 2.14	0.627	0.269	0.031
Sex (male) (%)	49.2%	43.3%	38.3%	0.601	0.619	0.168
Type of AF (parox)	55.6%	53.3%	48%	0.843	0.613	0.351
Duration of AF (months)	60.8 ± 22.25	38.68 ± 23.32	45.00 ± 10.27	0.192	0.569	0.142
BMI (kg/m^2^)	30.3 ± 1.86	29.26 ± 1.99	29.71 ± 1.28	0.454	0.717	0.565
LVEF (%)	57.9 ± 3.20	57.05 ± 7.20	59.44 ± 2.56	0.800	0.434	0.449
LA size (mm)	58.2 ± 2.46	55.38 ± 3.85	58.88 ± 2.04	0.226	0.108	0.649

**Table 2:** Procedural characteristics.

Parameter	LPLD RF (63)	HPSD RF (30)	CB (102)	*p*-Value (LPLD vs. HPSD)	*p*-Value (HPSD vs. CB)	*p*-Value (LPLD vs. CB)
Proc. time (min)	130.4 ± 8.21	142.07 ± 15.83	136.21 ± 7.39	0.147	0.467	0.310
Ablation time (min)	96.2 ± 7.64	103.93 ± 13.92	97.01 ± 6.75	0.289	0.343	0.877
X-ray time (min)	1.5 ± 1.28	6.03 ± 7.64	7.54 ± 0.78	0.555	0.456	0.966
X-ray dose (Gy)	554.7 ± 202.79	410.17 ± 168.94	355.80 ± 89.38	0.363	0.566	0.044

**Table 3:** Remapping outcomes.

Parameter	LPLD RF (63)	HPSD RF (30)	CB (102)	*p*-Value (LPLD vs. HPSD)	*p*-Value (HPSD vs. CB)	*p*-Value (LPLD vs. CB)
All veins isolated	0 (0%)	7 (23.3%)	11 (10.1%)	0.000	0.079	0.007
N. of reconnected segments	8.3 ± 1.08	4.47 ± 1.54	6.00 ± 0.91	0.000	0.091	0.001
N. of reconnected veins	2.7 ± 0.27	1.77 ± 0.48	2.36 ± 0.26	0.000	0.033	0.068
Most common reconnected segment	right carina	LSPV ridge/ant	LSPV ridge/ant	-	-	-
Most common reconnected vein	RIPV	LSPV	LSPV	-	-	-

## Data Availability

The data that support the findings of this study are available from the corresponding author upon reasonable request.

## Supplementary Materials

The following supporting information can be downloaded at: https://www.mdpi.com/article/10.3390/jcdd12080298/s1, Figure S1: Bipolar voltage remap of a patient with recurrent atrial fibrillation following index high-power short-duration (HPSD) radiofrequency (RF) pulmonary vein isolation (PVI). (a) No pulmonary vein reconnection (PVR) detected during the remap before redo ablation.; (b) Posterior wall debulking was performed using very high-power RF applications (90 W for 4 seconds). Abbre-viations: HPSD, high-power, short-duration; RF, radiofrequency; PVI, pulmonary vein isolation; PVR, pulmonary vein reconnection; Figure S2: Pulmonary Vein Reconnection (PVR) gap locations according to atrial fibrillation type: comparison of PVR gap distribution in patients with paroxysmal versus persistent atrial fibrillation; Figure S3: Twelve-month arrhythmia-free survival following redo PVI in patients with paroxysmal vs. persistent atrial fibrillation; Figure S4: Multivariate logistic regression analysis of predictors of pulmonary vein reconnection: initial ablation technique emerged as the only independent predictor of pulmonary vein reconnection; Figure S5: Effect of initial ablation modality on Pulmonary Vein Isolation (PVI) outcomes: comparison of clinical outcomes based on the initial ablation technique: low-power, long-duration radiofrequency (LPLD RF); high-power, short-duration radiofrequency (HPSD RF); and cryoballoon (CB) ablation; Table S1: Comparison of redo success rates in patients with and without pulmonary vein reconnection (PVR) following initial pulmonary vein isolation (PVI) using low-power, long-duration radiofrequency (LPLD RF); high-power, short-duration radiofrequency (HPSD RF); and cryoballoon (CB) ablation; Table S2: Baseline demographic and procedural characteristics of patients with and without pulmonary vein reconnection (PVR) following initial pulmonary vein isolation (PVI). Statistical comparisons were performed to assess differences between the PVR and no-PVR groups; Table S3: Baseline demographic and procedural characteristics of patients presenting with atrial fibrillation (AF) versus organized atrial tachycardia (AT) as the recurrent arrhythmia following initial pulmonary vein isolation (PVI). Comparisons were made to identify factors associated with the type of arrhythmia recurrence; Table S4: Baseline demographic procedural characteristics of patients presenting with paroxysmal versus persistent atrial fibrillation (AF) at the time of the index pulmonary vein isolation (PVI). Comparisons were made to evaluate differences between AF subtypes; Table S5: Procedural characteristics of patients presenting with paroxysmal versus persistent atrial fibrillation (AF) during the redo pulmonary vein isolation (PVI). Comparisons were made to evaluate differences between AF subtypes; Table S6: Remapping findings of patients presenting with paroxysmal versus persistent atrial fibrillation (AF) at the time of the remapping. Comparisons were made to assess differences in electrophysiological substrate between AF subtypes; Table S7: Twelve-month success rate of patients presenting with paroxysmal versus persistent atrial fibrillation (AF) following redo pulmonary vein isolation (PVI).

## Abbreviations

The following abbreviations are used in this manuscript:

AI	Ablation Index
AF	Atrial Fibrillation
AT	Atrial Tachycardia
BMI	Body Mass Index
CB	Cryoballoon
HPSD	High Power, Short Duration
LAA	Left Atrial Appendage
LA	Left Atrium
LIPV	Left Inferior Pulmonary Vein
LSPV	Left Superior Pulmonary Vein
LVEF	Left Ventricular Ejection Fraction
LPLD	Low Power, Long Duration
PV	Pulmonary Vein
PVI	Pulmonary Vein Isolation
PVR	Pulmonary Vein Reconnection
PFA	Pulsed Field Ablation
RF	Radiofrequency
RIPV	Right Inferior Pulmonary Vein
RSPV	Right Superior Pulmonary Vein
