# Supplementary material for: Electrophysiological Substrate and Pulmonary Vein Reconnection Patterns in Recurrent Atrial Fibrillation: Comparing Thermal Strategies in Patients Undergoing Redo Ablation
Source: J Cardiovasc Dev Dis. 2025 Aug 2;12(8):298. doi: 10.3390/jcdd12080298 (PMC12386683; doi:10.3390/jcdd12080298)
Supplement: Supplementary file 1 [file jcdd-12-00298-s001.zip › jcdd-3733404-supplementary.pdf]

Supplementary figures with legends

Supplementary Figure S1

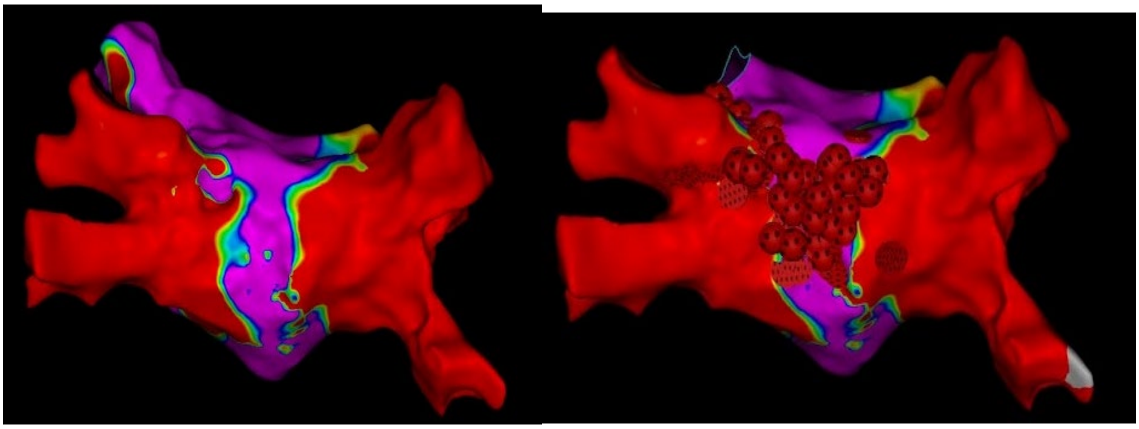

Figure S1: Bipolar voltage remap of a patient with recurrent atrial fibrillation following index high-power short-duration (HPSD) radiofrequency (RF) pulmonary vein isolation (PVI). (a) No pulmonary vein reconnection (PVR) detected during the remap before redo ablation. (b) Posterior wall debulking was performed using very high-power RF applications (90 W for 4 seconds). Abbreviations: HPSD, high-power, short-duration; RF, radiofrequency; PVI, pulmonary vein isolation; PVR, pulmonary vein reconnection.

Supplementary Figure S2

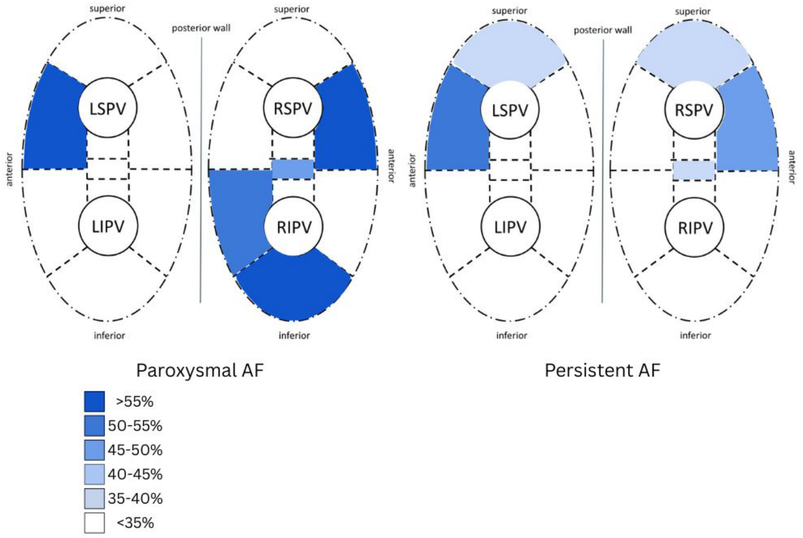

Figure S2: Pulmonary Vein Reconnection (PVR) gap locations according to atrial fibrillation type: comparison of PVR gap distribution in patients with paroxysmal versus persistent atrial fibrillation.

Supplementary Figure S3

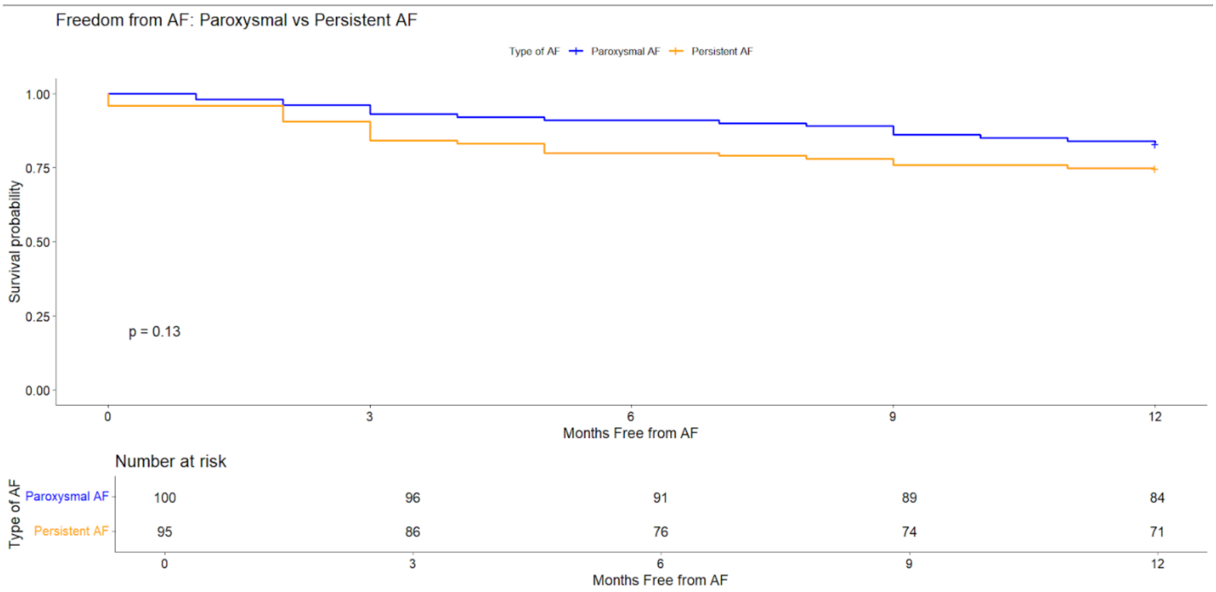

Figure S3: Twelve-month arrhythmia-free survival following redo PVI in patients with paroxysmal vs. persistent atrial fibrillation

Supplementary Figure S4

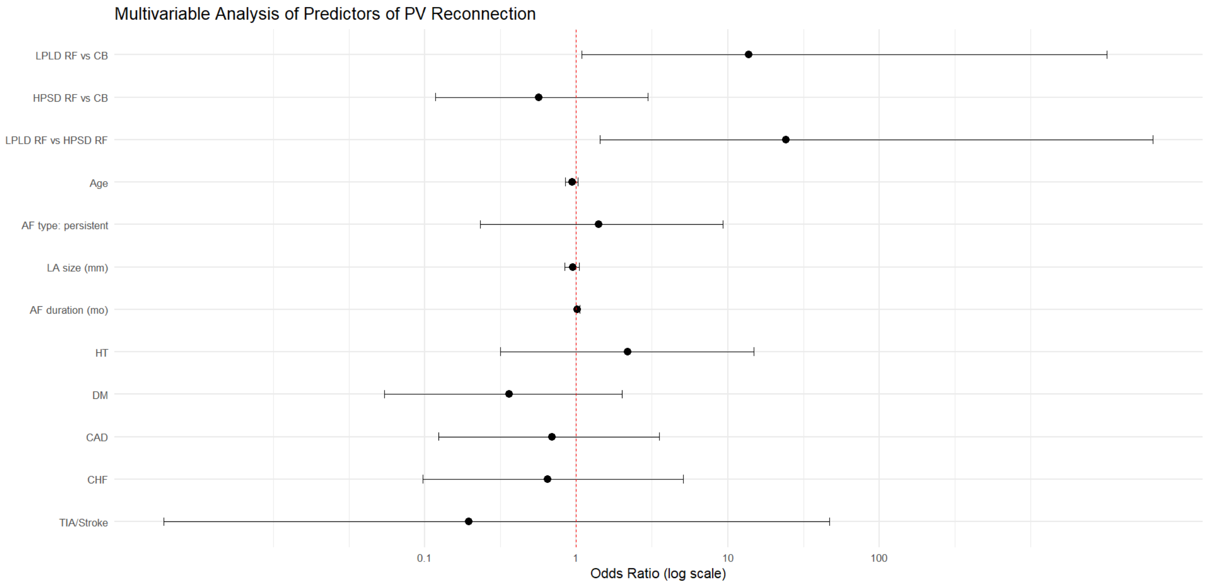

Figure S4: Multivariate logistic regression analysis of predictors of pulmonary vein reconnection: initial ablation technique emerged as the only independent predictor of pulmonary vein reconnection.

Supplementary Figure S5

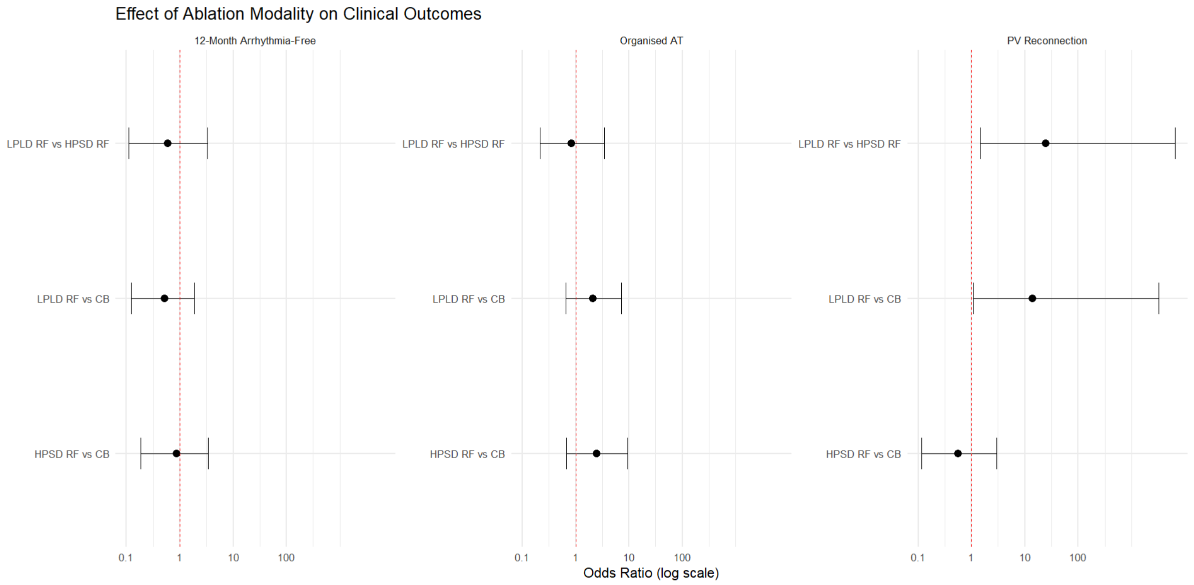

Figure S5: Effect of initial ablation modality on Pulmonary Vein Isolation (PVI) outcomes: comparison of clinical outcomes based on the initial ablation technique: low-power, long-duration radiofrequency (LPLD RF); high-power, short-duration radiofrequency (HPSD RF); and cryoballoon (CB) ablation.

Supplementary Table S1

		LPLD RF	HPSD RF	CB
No PVR (isolated)	no. of cases	0	7	11
	redo success rate	-	100% (7)	63.6% (7)
PVR	no. of cases	63	23	91
	redo success rate	77.7% (49)	86.9% (20)	79.1% (72)

Table S1. Comparison of redo success rates in patients with and without pulmonary vein reconnection (PVR) following initial pulmonary vein isolation (PVI) using low-power, long-duration radiofrequency (LPLD RF); high-power, short-duration radiofrequency (HPSD RF); and cryoballoon (CB) ablation.

Supplementary Table S2

	PVR (177)	No PVR (18)	p-value
Age	62.82 ± 1.61	69.89 ± 4.42	0.008
Sex (male)	104 (58.8%)	8(44.4%)	0.244
Duration of AF (month)	51.05 ± 9.96	23.30 ± 10.64	0.089
Type of AF (parox)	94 (53.2%)	6 (33.3%)	0.111
BMI	29.94 ± 0.98	28.83 ± 3.01	0.483
HT	131 (74.0%)	15 (83.3%)	0.388
DM	38 (21.5%)	5 (27.8%)	0.541
CAD	63 (35.6%)	8 (44.4%)	0.46
CHF	14 (7.9%)	3 (16.7%)	0.212
TIA/Stroke	10 (5.6%)	1 (5.6%)	0.987
Apnea	3 (1.7%)	3 (16.7%)	0
COPD	14 (7.9%)	2 (11.1%)	0.639
CHA2DS2-VASc	2.53 ± 0.20	3.44 ± 0.80	0.009
Procedure time (mins)	133.64 ± 5.40	150.67 ± 19.31	0.061
Ablation time (mins)	96.37 ± 4.89	111.94 ± 18.31	0.06
X-ray time (mins)	7.50 ± 0.70	7.82 ± 2.79	0.817
X-ray dose (Gy)	448.43 ± 91.56	231.61 ± 106.96	0.141
Redo timing	10.83 ± 1.78	11.22 ± 2.96	0.892
Complication	4 (2.3%)	0 (0.0%)	0.522
non-AF post PVI tachycardia	43 (24.3%)	6 (33.3%)	0.3996
12 month redo success rate	140 (79.1%)	14 (77.8%)	0.896

Table S2. Baseline demographic and procedural characteristics of patients with and without pulmonary vein reconnection (PVR) following initial pulmonary vein isolation (PVI). Statistical comparisons were performed to assess differences between the PVR and no-PVR groups.

Supplementary Table S3

	AF as recurrent arrhythmia (146)	Organized AT (49)	p-value
Age	62.90 ± 1.76	65.16 ± 3.20	0.209
Sex (male)	57 (39.0%)	26 (53.1%)	0.087
Duration of AF (month)	50.67 ± 11.30	41.28 ± 13.43	0.403
Type of AF (parox)	78 (53.4%)	22 (44.9%)	0.304
BMI	29.74 ± 1.15	30.06 ± 1.56	0.756
HT	113 (77.4%)	33 (67.3%)	0.162
DM	32 (21.9%)	11 (22.4%)	0.939
CAD	55 (37.7%)	16 (32.7%)	0.53
CHF	11 (7.5%)	6 (12.2%)	0.314
TIA/Stroke	7 (4.8%)	4 (8.2%)	0.379
Apnea	5 (3.4%)	1 (2.0%)	0.63
COPD	13 (8.9%)	3 (6.1%)	0.542
CHA2DS2-VASc	2.53 ± 0.23	2.86 ± 0.40	0.167
Procedure time (mins)	133.26 ± 5.78	141.04 ± 11.76	0.201
Ablation time (mins)	95.83 ± 5.50	103.71 ± 9.42	0.155
X-ray time (mins)	7.42 ± 0.79	7.83 ± 1.26	0.609
X-ray dose (Gy)	419.13 ± 98.78	456.10 ± 164.16	0.708
Redo timing	10.56 ± 1.83	11.78 ± 3.66	0.526
Complication	4 (2.7%)	0 (0.0%)	0.244
12 month redo success rate	118 (80.8%)	36 (73.5%)	0.2744

Table S3. Baseline demographic and procedural characteristics of patients presenting with atrial fibrillation (AF) versus organized atrial tachycardia (AT) as the recurrent arrhythmia following initial pulmonary vein isolation (PVI). Comparisons were made to identify factors associated with the type of arrhythmia recurrence.

Supplementary Table S4

Variable	Paroxysmal (n = 100)	Persistent (n = 95)	p-value
Age (years)	62.9 ± 0.8	63.22 ± 2.30	0.754
Sex (male)	46% (46)	69.5% (66)	0.001
Duration of AF (mo)	52.9 ± 1.4	63.4 ± 2.20	0.001
BMI	29.6 ± 1.1	30.3 ± 1.57	0.661
LVEF (%)	62.3 ± 1.78	54.87 ± 3.27	0.006
LA size (mm)	55.7 ± 2.02	60.32 ± 1.93	0.008
HT	73% (73)	76.8% (73)	0.598
DM	21% (21)	23.2% (22)	0.693
CAD	33% (33)	40% (38)	0.343
CHF	0% (0)	17.9% (17)	<0.001
TIA/stroke	5% (5)	6.3% (6)	0.682
Apnea	1% (1)	5.3% (5)	0.096
COPD	1% (1)	11.6% (11)	0.003
CHA2DS2-VASc	2.6 ± 0.26	2.6 ± 0.64	0.798
Antiarrhythmics	95% (95)	100% (95)	0.027
Anticoagulation	94% (94)	95.8% (91)	0.574

Table S4. Baseline demographic procedural characteristics of patients presenting with paroxysmal versus persistent atrial fibrillation (AF) at the time of the index pulmonary vein isolation (PVI). Comparisons were made to evaluate differences between AF subtypes.

Supplementary Table S5

Variable	Paroxysmal (n = 100)	Persistent (n = 95)	p-value
Procedure time (min)	135.6 ± 7.58	134.79 ± 7.26	0.900
Ablation time (min)	66.3 ± 8.69	97.85 ± 9.3	<0.001
X-ray time (min)	6.6 ± 0.89	6.8 ± 0.87	0.522
X-ray dose (Gy)	317.2 ± 73.43	383.6 ± 152.24	0.285
Complication	3% (3)	1.1% (1)	0.361
Timing of redo (mo)	10.2 ± 1.78	11.59 ± 2.81	0.396

Table S5. Procedural characteristics of patients presenting with paroxysmal versus persistent atrial fibrillation (AF) during the redo pulmonary vein isolation (PVI). Comparisons were made to evaluate differences between AF subtypes.

Supplementary Table S6

Variable	Paroxysmal (n = 100)	Persistent (n = 95)	p-value
All veins isolated	6% (6)	12.6% (12)	0.111
Number of reconnected veins	7.0 ± 0.89	5.96 ± 0.9	0.094
Number of reconnected segments	2.5 ± 0.23	2.3 ± 0.22	0.182
Most common reconnection site	RIPV	LSPV ant/ridge	–
Most common non-AF post-PV trigger	22% (22)	28.4% (27)	0.304

Table S6. Remapping findings of patients presenting with paroxysmal versus persistent atrial fibrillation (AF) at the time of the remapping. Comparisons were made to assess differences in electrophysiological substrate between AF subtypes.

Supplementary Table S7

Variable	Paroxysmal (n = 100)	Persistent (n = 95)	p-value
Redo 12-month success	83% (83)	71.74% (74)	0.215

Table S7. Twelve-month success rate of patients presenting with paroxysmal versus persistent atrial fibrillation (AF) following redo pulmonary vein isolation (PVI).

multivariate analysis

```
logistf(formula = reconnection ~ ablation_modality + Age + AF_type +
  `LA size (mm)` + `Duration of AF (month)` + HT + DM + CAD +
  CHF + `TIA/Stroke`, data = df)

Model fitted by Penalized ML
Coefficients:
                      coef   se(coef)   lower 0.95  upper 0.95
(Intercept)            7.99252459 3.57995718  0.564325303 17.82024981
ablation_modalityHPSD_RF -0.56729432 0.73075804 -2.143743197  1.08963149
ablation_modalityLPLD_RF  2.62688478 1.35037536  0.081673294  8.07204132
Age                  -0.06241291 0.03994608 -0.164171456  0.02171591
AF_typepersistent       0.34127640 0.80153951 -1.463273103  2.22679567
`LA size (mm)`          -0.05569063 0.04418077 -0.172404422  0.04412565
`Duration of AF (month)`  0.01789091 0.01060563 -0.003558148  0.05433144
HT                    0.78301353 0.83977683 -1.151866870  2.70172439
DM                   -1.02355059 0.76672787 -2.922326129  0.69479203
CAD                   -0.36857361 0.72686646 -2.096506725  1.26158617
CHF                   -0.43298641 0.89253301 -2.337102208  1.62400507
`TIA/Stroke`           -1.63983293 1.76869489 -6.273405680  3.85327869
                     Chisq        p method
(Intercept)           4.4953349 0.03398746      2
ablation_modalityHPSD_RF 0.4916307 0.48320078      2
ablation_modalityLPLD_RF 4.1607600 0.04137084      2
Age                  2.0679668 0.15042194      2
AF_typepersistent       0.1433817 0.70494198      2
`LA size (mm)`          1.1748641 0.27840405      2
`Duration of AF (month)`  2.5225800 0.11222683      2
```

HT 0.6880646 0.40682348 2

DM 1.4012064 0.23652168 2

CAD 0.2029545 0.65234658 2

CHF 0.1996490 0.65500434 2

`TIA/Stroke` 0.5346075 0.46467620 2

Method: 1-Wald, 2-Profile penalized log-likelihood, 3-None

Likelihood ratio test = 11.53809 on 11 df, p = 0.3993448, n = 83

Wald test = 25.813 on 11 df, p = 0.006918662>

=> A multivariable logistic regression analysis using Firth's penalized likelihood method was performed to evaluate whether ablation modality independently predicted pulmonary vein (PV) reconnection, adjusting for age, AF type, left atrial (LA) size, AF duration, and key comorbidities.

The overall model was statistically significant based on the Wald test (χ^2 = 25.81, *p* = 0.0069), though the likelihood ratio test was not significant (χ^2 = 11.54, *p* = 0.399).

Among the predictors, ablation modality was significantly associated with PV reconnection:

- Patients who underwent LPLD RF ablation had significantly higher odds of PV reconnection compared to those treated with cryoballoon (CB) ablation (β = 2.63, 95% CI: 0.08 to 8.07, *p* = 0.041).
    - This corresponds to an odds ratio (OR) of approximately 13.83 (exp(2.63)), suggesting a strong association between LPLD RF and reconnection.
- The difference between HPSD RF and CB ablation was not statistically significant (β = -0.57, 95% CI: -2.14 to 1.09, *p* = 0.483).

None of the clinical covariates—including age, AF type (persistent vs. paroxysmal), LA size, AF duration, or comorbidities (HT, DM, CAD, CHF, prior TIA/stroke)—were significantly associated with PV reconnection in this model.

```
> summary(pv_model)
logistf(formula = reconnection ~ ablation_modality + Age + AF_type +
    LA_size + AF_duration + HT + DM + CAD + CHF + TIA_Stroke,
    data = df)

Model fitted by Penalized ML
Coefficients:
                                coef   se(coef)   lower 0.95  upper 0.95
(Intercept)               7.99252459 3.57995718  0.564325303 17.82024981
ablation_modalityHPSD_RF -0.56729432 0.73075804 -2.143743197  1.08963149
ablation_modalityLPLD_RF  2.62688478 1.35037536  0.081673294  8.07204132
Age                      -0.06241291 0.03994608 -0.164171456  0.02171591
AF_typepersistent         0.34127640 0.80153951 -1.463273103  2.22679567
LA_size                  -0.05569063 0.04418077 -0.172404422  0.04412565
AF_duration               0.01789091 0.01060563 -0.003558148  0.05433144
HT                        0.78301353 0.83977683 -1.151866870  2.70172439
DM                       -1.02355059 0.76672787 -2.922326129  0.69479203
CAD                      -0.36857361 0.72686646 -2.096506725  1.26158617
CHF                      -0.43298641 0.89253301 -2.337102208  1.62400507
TIA_Stroke               -1.63983293 1.76869489 -6.273405680  3.85327869
                             Chisq          p method
(Intercept)              4.4953349 0.03398746      2
ablation_modalityHPSD_RF 0.4916307 0.48320078      2
ablation_modalityLPLD_RF 4.1607600 0.04137084      2
Age                      2.0679668 0.15042194      2
AF_typepersistent        0.1433817 0.70494198      2
LA_size                  1.1748641 0.27840405      2
AF_duration              2.5225800 0.11222683      2
HT                       0.6880646 0.40682348      2
DM                       1.4012064 0.23652168      2
CAD                      0.2029545 0.65234658      2
CHF                      0.1996490 0.65500434      2
TIA_Stroke               0.5346075 0.46467620      2

Method: 1-Wald, 2-Profile penalized log-likelihood, 3-None

Likelihood ratio test=11.53809 on 11 df, p=0.3993448, n=83
Wald test = 25.813 on 11 df, p = 0.006918662> aa_model <- logistf(`AA duri
```

```
> nonAF_model <- logistf(`post PVI tachycardia` ~ ablation_modality + Age + AF_type +
+                     AF_duration + HT + DM + CAD + CHF + TIA_Stroke,
+                   data = df)
>  summary(nonAF_model)
logistf(formula = `post PVI tachycardia` ~ ablation_modality +
    Age + AF_type + LA_size + AF_duration + HT + DM + CAD + CHF +
    TIA_Stroke, data = df)

Model fitted by Penalized ML
Coefficients:
                                 coef    se(coef)  lower 0.95  upper 0.95
(Intercept)              -1.445549834 2.101383086 -5.87219234 2.754881945
ablation_modalityCB      -0.917165962 0.644733746 -2.24256658 0.401258298
ablation_modalityLPLD_RF -0.175690276 0.685219724 -1.55323084 1.241804119
Age                       0.032073729 0.027165298 -0.02134331 0.091393991
AF_typepersistent         0.879102807 0.587526252 -0.27967751 2.136746144
LA_size                  -0.006989184 0.031700571 -0.07451133 0.057834371
AF_duration              -0.003603971 0.005270959 -0.01590910 0.006525115
HT                       -1.058588289 0.647856123 -2.39423833 0.238853050
DM                       -0.463699813 0.648185927 -1.92962474 0.791536252
CAD                      -0.168737448 0.537721417 -1.29901798 0.910454952
CHF                      -0.226401827 0.767415639 -1.90081869 1.275586119
TIA_Stroke               -0.294788632 1.109459233 -2.86655139 1.855188866
                              Chisq         p method
(Intercept)              0.45314435 0.5008455      2
ablation_modalityCB      1.88441721 0.1698330      2
ablation_modalityLPLD_RF 0.06223476 0.8029981      2
Age                      1.35293820 0.2447651      2
AF_typepersistent        2.19205749 0.1387239      2
LA_size                  0.04423441 0.8334181      2
AF_duration              0.44705493 0.5037368      2
HT                       2.57042699 0.1088783      2
DM                       0.49326347 0.4824751      2
CAD                      0.09260816 0.7608872      2
CHF                      0.08258411 0.7738256      2
TIA_Stroke               0.06785891 0.7944801      2

Method: 1-Wald, 2-Profile penalized log-likelihood, 3-None

Likelihood ratio test=9.410388 on 11 df, p=0.5840675, n=83
Wald test = 20.03462 on 11 df, p = 0.04486816> |
```

A multivariable logistic regression model was used to assess whether ablation modality independently predicted the occurrence of non-AF atrial tachycardia following redo PVI. After adjusting for age, AF type, LA size, AF duration, and comorbidities (HT, DM, CAD, CHF, prior stroke/TIA), ablation modality was not significantly associated with the occurrence of post-PVI non-AF atrial tachycardia. None of the other covariates reached statistical significance either. The overall model was statistically significant by Wald test (p = 0.045), but not by likelihood ratio test (p = 0.58), suggesting potential instability in model estimates possibly due to small sample size or sparse event distribution.

```
Wald test = 18.78663 on 11 df, p = 0.0650328> survival_model <- logistf(`1 yea
+                              AF_duration + HT + DM + CAD + CHF + TIA_Stroke,
+                            data = df)
> summary(survival_model)
logistf(formula = `1 year FU after redo` ~ ablation_modality +
    Age + AF_type + LA_size + AF_duration + HT + DM + CAD + CHF +
    TIA_Stroke, data = df)

Model fitted by Penalized ML
Coefficients:
                                 coef   se(coef)    lower 0.95  upper 0.95
(Intercept)              -6.962104834 2.74721163 -13.557631276 -1.66585238
ablation_modalityHPSD_RF -0.146943288 0.69136328  -1.682360252  1.22412277
ablation_modalityLPLD_RF -0.662020932 0.64875094  -2.099474669  0.63979680
Age                       0.048545651 0.03019412  -0.010801733  0.11844258
AF_typepersistent         1.000376132 0.60349055  -0.204837493  2.29853546
LA_size                   0.043385699 0.03327950  -0.024770596  0.11627398
AF_duration               0.004693878 0.00514457  -0.006743127  0.01517736
HT                       -0.552233339 0.73697688  -2.064053978  1.05056435
DM                        0.307931830 0.61528105  -1.022596462  1.55566933
CAD                      -0.148371933 0.56447337  -1.353137567  1.01460420
CHF                      -0.719964095 0.82918076  -2.658432978  0.88050553
TIA_Stroke               -0.839952925 1.52488991  -5.815607747  1.75975991
                              Chisq           p method
(Intercept)              6.99574787 0.008170357      2
ablation_modalityHPSD_RF 0.04176456 0.838069216      2
ablation_modalityLPLD_RF 0.96817396 0.325136010      2
Age                      2.52026335 0.112391814      2
AF_typepersistent        2.63856470 0.104297732      2
LA_size                  1.55476011 0.212434093      2
AF_duration              0.71163463 0.398901686      2
HT                       0.49795551 0.480399832      2
DM                       0.22405567 0.635966925      2
CAD                      0.06273023 0.802231613      2
CHF                      0.73167367 0.392341225      2
TIA_Stroke               0.31930052 0.572028313      2

Method: 1-Wald, 2-Profile penalized log-likelihood, 3-None

Likelihood ratio test=10.34836 on 11 df, p=0.4993509, n=83
Wald test = 24.69968 on 11 df, p = 0.01008516> |
```

A multivariable logistic regression was performed to identify predictors of arrhythmia-free survival at 12 months following redo ablation. After adjusting for ablation modality, age, AF type, LA size, AF duration, and key comorbidities, no variable—including ablation modality—was found to be a statistically significant independent predictor. While the overall model was significant by Wald test (p = 0.010), the likelihood ratio test did not reach significance (p = 0.499), indicating some model instability or possible overfitting due to limited sample size.
